# **Sequestration of Pb(II) using channel-like porous spheres of carboxylated graphene oxide-incorporated cellulose acetate@**i**minodiacetic acid: optimization and mechanism study**

**DOI:** 10.1007/s11356-024-33185-1

**Published:** 2024-04-25

**Authors:** Eman M. Abd El-Monaem, Hassanien Gomaa, Ahmed M. Omer, Gehan M. El-Subruiti, Abdelazeem S. Eltaweil

**Affiliations:** 1 Chemistry Department, Faculty of Science, Alexandria University, Alexandria, Egypt; 2 Department of Chemistry, Faculty of Science, Al-Azhar University, Assiut, 71524 Egypt; 3 Polymer Materials Research Department, Advanced Technology and New Materials Research Institute (ATNMRI), City of Scientific Research and Technological Applications (SRTA-City), New Borg El-Arab City, P. O. Box: 21934, Alexandria, Egypt; 4 Department of Engineering, Faculty of Engineering and Technology, University of Technology and Applied Sciences, Ibra, Sultanate of Oman

**Keywords:** Cellulose acetate, Pb(II), Intra-particle diffusion, XPS, Surface functionalization

## Abstract

The adsorption property of the costless green cellulose acetate (CA) was boosted by the dual modifications: inner modification by incorporating carboxylated graphene oxide (COOH-GO) into the CA spheres and outer modification by the surface modification of the COOH-GO@CA spheres by iminodiacetic acid (IDA) for removing Pb(II). The adsorption experiments of the Pb(II) proceeded in a batch mode to evaluate the adsorption property of the COOH-GO@CA@IDA spheres. The maximal Pb(II) adsorption capacity attained 613.30 mg/g within 90 min at pH = 5. The removal of Pb(II) reached its equilibrium within 20 min, and the removal % was almost 100% after 30 min at the low Pb(II) concentration. The Pb(II) adsorption mechanism was proposed according to the kinetics and isotherms studies; in addition, the zeta potential (ZP) measurements and X-ray Photoelectron Spectroscopy (XPS) analysis defined the adsorption pathways. By comparing the XPS spectra of the authentic and used COOH-GO@CA@IDA, it was deduced that the contributed chemical adsorption pathways are Lewis acid–base, precipitation, and complexation. The zeta potential (ZP) measurements demonstrated the electrostatic interaction participation in adsorbing the cationic Pb(II) species onto the negatively charged spheres (ZP = 14.2 mV at pH = 5). The unique channel-like pores of the COOH-GO@CA@IDA spheres suggested the pore-filling mechanism of Pb(II). The promising adsorption results and the superb recyclability character of COOH-GO@CA@IDA enable it to extend of the bench scale to the industrial scale.

**Supplementary Information:**

The online version contains supplementary material available at 10.1007/s11356-024-33185-1.

## Introduction

Certainly, contamination of freshwater with heavy metals is a matter of global concern. Lead is ranked at the top of the list of the most detrimental heavy metals that dreadfully impact human organs, such as the circulatory system, brain, kidneys, and nervous system. According to the World Health Organization (WHO), the maximum allowable concentration of lead in drinking water should not exceed 0.05 mg/L. The Environmental Protection Agency (EPA) sets the permissible limit for Pb (II) in wastewater at 0.05 mg/L. However, industrial wastewaters often contain lead-ion concentrations ranging from 200 to 500 mg/L, significantly surpassing water quality standards. To comply with regulations, it is essential to reduce lead-ion concentrations in industrial wastewaters to a range of 0.05–0.10 mg/L before discharge into water bodies or sewage systems (Arbabi et al. 2015). Therefore, Scientists have pursued to find a viable strategy to remove the accumulated concentrations of lead in drinking water. Among the diverse manners that exhibited appropriate performance during lead removal are solvent extraction, electrochemical, membrane filtration, chemical oxidation, coagulation, and flocculation (Qasem et al. 2021; Wang et al. 2018). Nevertheless, the worthy features of the adsorption, including energy saving, reasonable capital cost, remarkable removal performance, and facile processing, render it the preeminent choice for controlling the hazard of such toxic species in drinking water (Abd El-Monaem et al. 2022a; Osman et al. 2023; Sheikh et al. 2023), or this sake, varied substances like carbon-based materials, metal hydroxides (Peng et al. 2005), clays, and polymers (Omer et al. 2023b; Shen et al. 2022) have been fostered to meet the criteria of efficacious adsorbents.

The recent global situation of widespread pollution, climate change, and the resources lack turn the focal point to exploit eco-friendly natural resources in the different sectors (Eltaweil et al. 2023b). Using such natural adsorbents declines the chemical manufacturing of the adsorbents that result in dangerous fumes. In addition, the fabrication of many adsorbents involves the utilization of harmful solvents, such as dimethyl formamide, which is one of the main components for the preparation of metal–organic frameworks. In particular, green adsorbents like natural polymers have drawn striking attention in drinking water purification for their outstanding efficiency, natural profusion, prime recyclability, and non-toxicity. Among the cellulose derivatives, cellulose acetate (CA) has been exploited in many fields; batteries, food packaging, catalysis, and gas storage (Abd El-Monaem et al. 2022b). Cellulose acetate has revealed promising results when used as an adsorbent for drinking water purification. Thus, some recent studies have been performed to enhance the adsorption performance of CA, such as Eltaweil et al. study that involved encapsulating high-capacity substance like GO into the CA matrix for removing methylene blue, revealing an enhancement in the adsorption capacity of CA from 77.22 to 157.96 mg/g after the incorporation of GO (Eltaweil et al. 2023a). Furthermore, the surface functionality of CA by active adsorption species; for instance, Chen et al. modified the surface of CA by deacetylation, carboxymethylation, and polydopamine coating, forming a developed adsorbent with high adsorption aptitude toward the cationic methylene blue (69.89 mg/g) and the anionic Congo red (67.31 mg/g) (Chen et al. 2020). Additionally, few studies involved the applications of these two techniques, attaining promising results during adsorbing persistent contaminants. In this context, Omer et al. modified the CA beads by incorporating the amine-functionalized GO into the beads, and the surface was enriched by extra amine groups to prepare efficacious adsorbent with super-high adsorption capacity toward Cr(VI) attained 410.21 mg/g (Omer et al. 2022).

Graphene oxide (GO) is reticular of hexagonal structures that compose of epoxy and hydroxyl in the basal plane, in addition to the located alkyl and carboxyl in the sheets’ edges (Korkmaz &Kariper 2020). Hummer’s approach is the common strategy to fabricate GO by graphite oxidation. Graphene oxide has exceptional advantages, comprising a huge surface area, ample oxygenated functional groups, superior adsorption aptitude, and excellent mechanical properties (Yu et al. 2020). Thanks to the easy functionalization of GO that facilitates boosting its adsorption property by immobilizing an active site like carboxyl, amine, or sulfate groups to the GO structure (Razaq et al. 2022). Notably, the delocalized *π* electrons of GO enable it to be an outstanding adsorbent for removing noxious contaminants (Omer et al. 2022). Interestingly, encapsuling of GO into the polymer chain improves its characteristics: flexibility, surface area, thermal and mechanical properties, and adsorption efficacy. Nonetheless, the separation of GO/polymer adsorbents is difficult, which declines their recyclability. Molding of GO/polymer composites in the shapes like spheres, fibers, and membranes could provide perfect and easy separation.

This work intends to prepare an eco-friendly, highly efficient, and recyclable adsorbent for lead removal from aqueous media. The study provides a modified porous adsorbent with anionic functional groups inside the core and on the surface to enhance its adsorbability toward the cationic Pb(II) species. The inner- and outer-modification techniques were adopted to boost the adsorption property of cellulose acetate as follows; the inner-modification proceeded by optimizing the appropriate encapsulated proportion of carboxyl-functionalized GO into the CA spheres. Next, the outer-modification of the fabricated COOH-GO@CA was performed by attaching iminodiacetic acid to the spheres’ surface, forming COOH-GO@CA@IDA. Several analysis tools were employed to scrutinize the physiochemical, thermal, and morphological characteristics of COOH-GO@CA@COOH. Optimization of the predominant parameters of the Pb(II) adsorption onto COOH-GO@CA@IDA was executed in a batch mode. The Pb(II) adsorption mechanism onto COOH-GO@CA@IDA was presumed based on the kinetic and isotherm studies and the XPS analysis.

## Fabrication and application experiments

### The used chemicals

Text S1 compiled the specifications of the used chemicals in the experimental work.

### Preparation of COOH@GO

Firstly, GO was synthesized by modified Hummer’s approach as described in the authors’ previous work (Omer et al. 2023a). In detail, graphite powder (2 g) was dispersed under constant stirring at 4 °C in a mixture of 35 mL of concentrated HNO_3_ and 110 mL of concentrated H_2_SO_4_. Then, 12 g of KMnO_4_ (acts as oxidizing agent) was slowly added, while the reaction mixture was kept in an ultrasonic water bath for another 1 h. To complete the oxidation process, the mixture was continued stirred for 45 min at 40 °C, before emptying it in iced water (300 mL) under stirring. To terminate the reaction, H_2_O_2_ (20 mL, 30%) was added slowly, while the resultant brown product was settled overnight. The acquired GO was washed with HCl (10%) and then with distilled water until reached pH 6, and finally dried at 50 °C in an oven. Secondly, the carboxyl functionality of GO was prepared following the reported work (Eltaweil et al. 2020b). An accurate 10 mg of GO was dispersed in 50 mL of double-distilled H_2_O under sonication for 1 h. A total of 2.5 g of NaOH was dipped into the GO suspension, followed by adding 2.5 g of ClCH_2_COOH (implied for the conversion of -OH groups of GO into ether bonds (C–O–CH_2_COOH)) with continuous sonication for 2 h. Next, the pH medium of the formed COOH-functionalized GO was neutralized using HCl. Ultimately, the dark black particles of COOH-GO were separated, washed well several times by double distilled H_2_O and then by CH_3_OH, and dried at 65 °C for 10 h.

### Fabrication of COOH-GO@CA@IDA

COOH-GO@CA spheres were prepared by dissolving 1 g of CA into DMSO under potent stirring for 1 h. Next, varied proportions of COOH-GO at the range of 10–25 wt% were separately added to CA solutions under stirring for another 1 h to obtain an utterly homogeneous solution. The COOH-GO/CA solution was dropped by a syringe into a container containing distilled water as a coagulant. The formed spheres were kept in the coagulation medium for curing under slow stirring. After complete maturing to the spheres, they were collected and washed with distilled water to remove the residual DMSO (Eltaweil et al. 2023a; Omer et al. 2022). Then, after, the COOH-GO@CA surface was modified by soaking into PBQ with adjusting the pH solution to be 10 to activate the surface of spheres. After 1 h, COOH-GO@CA-PBQ spheres were separated and washed repeatedly with distilled H_2_O. COOH-GO@CA-PBQ spheres were dipped into an aqueous IDA solution (0.2 M) under gentle stirring for 1 h. Finally, COOH-GO@CA@IDA spheres were gathered and washed with distilled H_2_O to remove the unreacted IDA.

### Characterization instruments

Text S2 summarized the used instruments in characterizing the COOH-GO@CA@IDA spheres and the authentic components.

### Application experiments

The COOH-GO@CA@IDA spheres were applied in adsorbing Pb(II) species from an aqueous medium. Optimization parameters proceeded throughout a series of lab experiments. A comparison test was conducted to determine the appropriate encapsulated COOH-GO into the spheres. For identifying the optimal pH, the adsorption experiment was performed at pHs 3, 5, 7, 9, and 11. The economical dosage of COOH-GO@CA@IDA was determined after testing the removal aptitude of Pb(II) by varied dosages (5, 10, 15, and 20 mg). Moreover, the thermodynamics of the Pb(II)–COOH-GO@CA@IDA system was deduced by proceeding with the Pb(II) removal process at temperatures of 25, 35, 45, and 55 °C. Furthermore, the efficacy of COOH-GO@CA@IDA spheres to adsorb low and high concentrations of Pb(II) ions ranging from 50 to 450 mg/L. After contact time, the concentration of the Pb(II) species was measured by atomic absorption, and then the removal efficacy and the adsorption capacity were reckoned from Eqs. 1 and 2 (Abdelmonem et al. 2024; Naggar et al. 2023).

1 $$R (\mathrm{\%})=\frac{{C}_{o }- {C}_{t}}{{C}_{o}} \times 100$$

2 $${q}_{\mathrm{t }}({\text{mg}}/{\text{g}})=\frac{{(C}_{{\text{o}}}- {C}_{{\text{t}}}) \times V}{m}$$

where R% clarifies the percent of the removed pollutant from the treatment system. *q*_t_ represents the amount of the adsorbed pollutant per the mass of adsorbent. The initial concentration of Pb(II) is *C*_o_, and the concentration of Pb(II) after the purification process is *C*_t_. The amount of the used COOH-GO@CA@IDA is symbolized as *m*, and *V* represents the volume of Pb(II).

## Results and discussion

### Characterization analyses

#### SEM

**Fig. 1:**
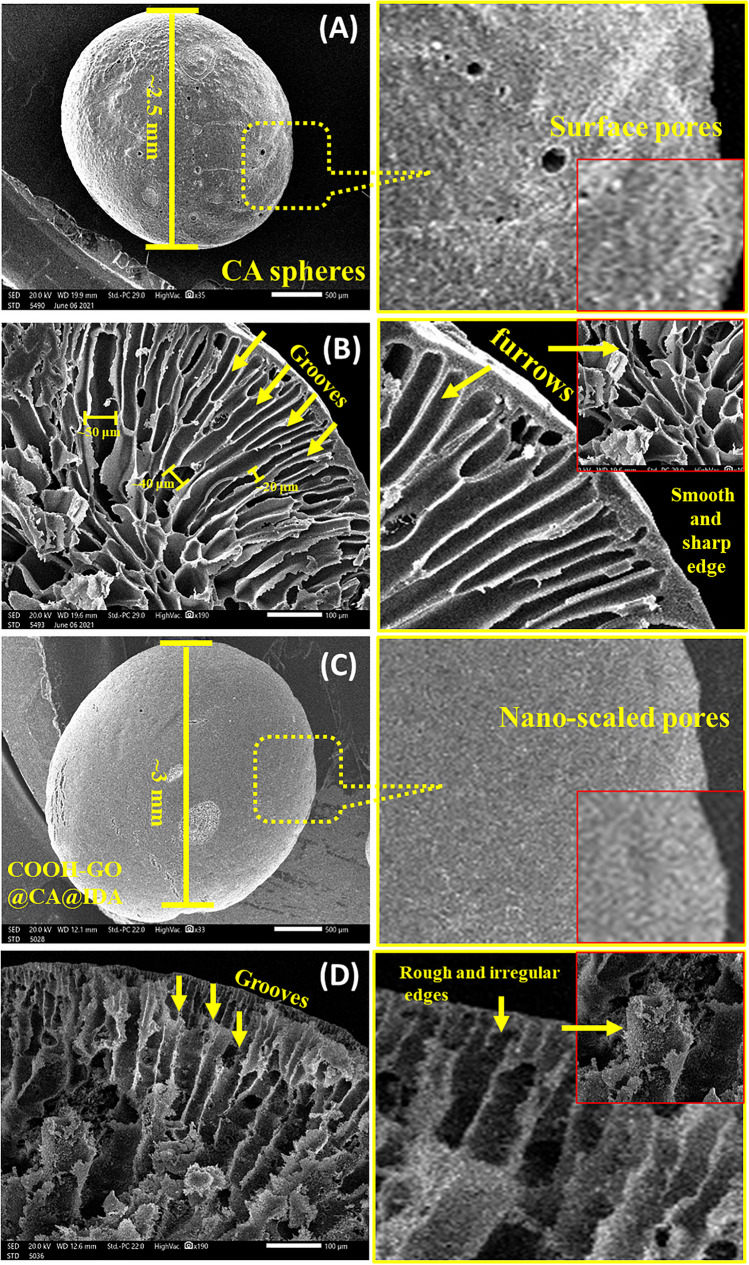
**A**, **B** SEM images of CA spheres and **C**, **D** COOH-GO@CA@IDA samples

Figure 1A–D show the top-views SEM images of CA, and COOH-GO@CA@IDA spheres samples with different magnifications, in both surface and cross-section. From Fig. 1A and C, we can notice obviously that the diameter of CA, and COOH-GO@CA@IDA spheres are about 2.5–3 mm. The high-magnification SEM image of the CA spheres shows essentially the smooth surface morphology composed of dispersed apertures with a pore size > 50 nm, as shown in Fig. 1A. In the cross-sectional image of the CA spheres, there are long parallel grooves and furrows (width 20–50 µm) expanding from the surface to the core of the spheres. These grooves have a smooth and sharp edge, as explained in Fig. 1B. These micro-sized grooves resembling caves may efficiently contribute significantly to the trapping of the target ions. In contrast, the top-view SEM image of COOH-GO@CA@IDA spheres **(**Fig. 1C**)** display a different morphology, where the surface became smoother and softness with lower pore size (< 50 nm). The cross-sectional SEM image of the COOH-GO@CA@IDA spheres was also taken, as displayed in Fig. 1D. Where the inside-core morphology of the cross-section shows the channel-like pores of the spheres with rough and irregular edges owes to the incorporation of COOH-GO. The Figure S1 depicted the sheet-like morphology of COOH-GO. The cross-sectional images of CA, and COOH-GO@CA@IDA spheres samples shows a hierarchical porous structure, a thin outer mantle of 10 µm in thickness, and a thick inner groove edge of < 0.5 µm in thickness. The distinctive morphology of COOH-GO@CA@IDA spheres with grooves, furrows, and surface pores plays a considerable role in the trapping/adsorption of Pb(II) ions from aqueous solutions. This distinguished structure may enhance the diffusion of Pb(II) ions through multi grooves and furrows, adsorption efficiency, and fast adsorption-elution processes.

#### FTIR

**Fig. 2:**
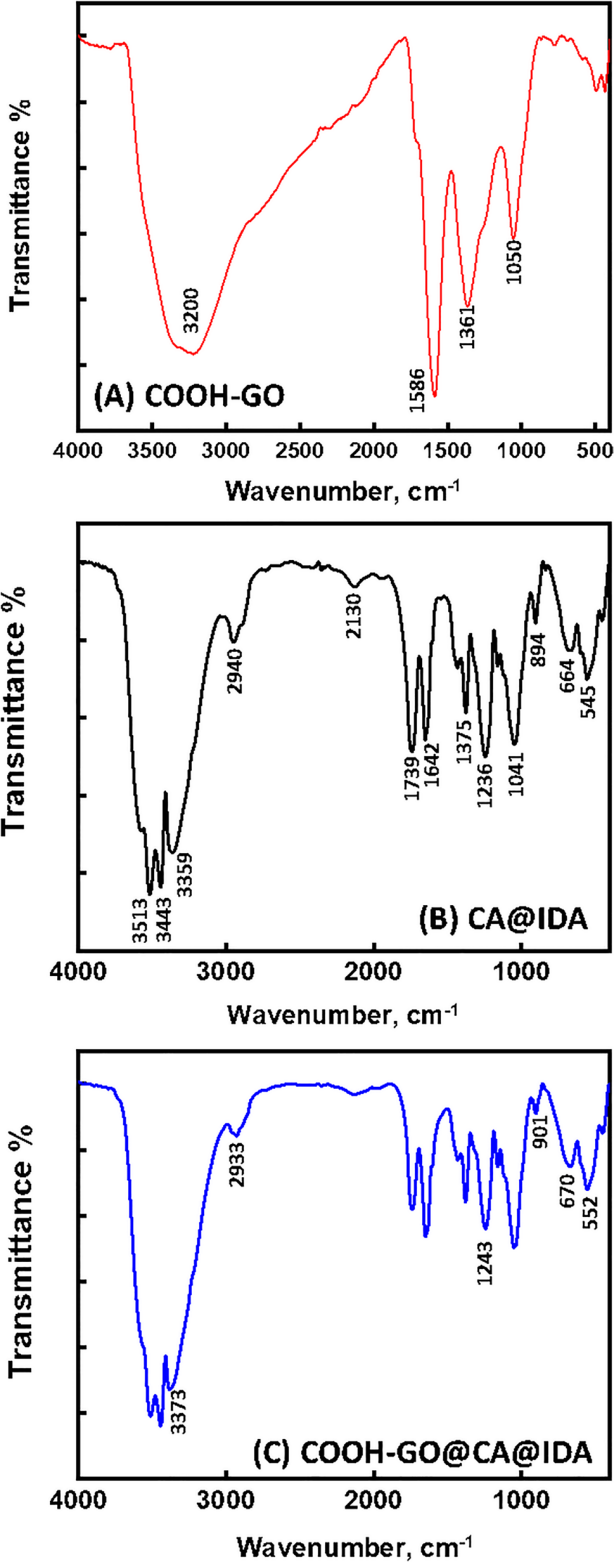
FTIR spectra of COOH-GO, CA@IDA, and COOH-GO@CA@IDA spheres

Figure 2 shows the FTIR spectra of COOH-GO, CA@IDA, and COOH-GO@CA@IDA. The FTIR spectra show that there were numerous absorption peaks in the range 4000–400 cm^−1^. Figure 2A of COOH-GO sample demonstrates a characteristic band at around 3200 cm^−1^ attributed to (− OH) stretching vibration of the –COOH groups. The peak in the low frequency region close to 1586 cm^−1^ is attributable to O–H vibrations of water. Corresponding peak for C–OH functional group can be clearly observed at 1371 cm^−1^. The peak corresponding to epoxy C–O–C (alkoxy) stretching vibration was identified at 1050 cm^−1^ (Andrijanto et al. 2016; He et al. 2015). Figure 2B shows the FTIR spectrum of CA@IDA, where the widening of absorption peaks was generally observed in the region of 3300–3550 cm^−1^ corresponding to the stretching of intermolecular hydrogen bonds of hydroxyl groups (-OH). The absorption peak at wavenumber of 2940 cm^−1^ corresponded to the stretching of –CH- of methyl groups (-CH_3_). The carbonyl group (C = O) stretching vibration was observed at 1739 cm^−1^. The band at 1375 cm^−1^ is designated C-O–H bending vibration. The characteristic peak of C–O–C antisymmetric stretching vibration of ester group of pure cellulose acetate was assigned at 1236 cm^−1^. Furthermore, the -C–OH stretching vibration of CACOOH was assigned at wavenumber of 1041 cm^−1^. The presence of the absorption peak at 894 cm^−1^ could be due to the combination of –C-O- stretching and -CH_2_- rocking vibrations (Sudiarti et al. 2017). The changes in the absorption peaks in terms of the wavenumber shifting relative intensity, disappearance of the existing peak and even appearance of new peaks evidence the formation of COOH-GO@CA@IDA spheres composite, as shown in Fig. 2C.

#### XRD

**Fig. 3:**
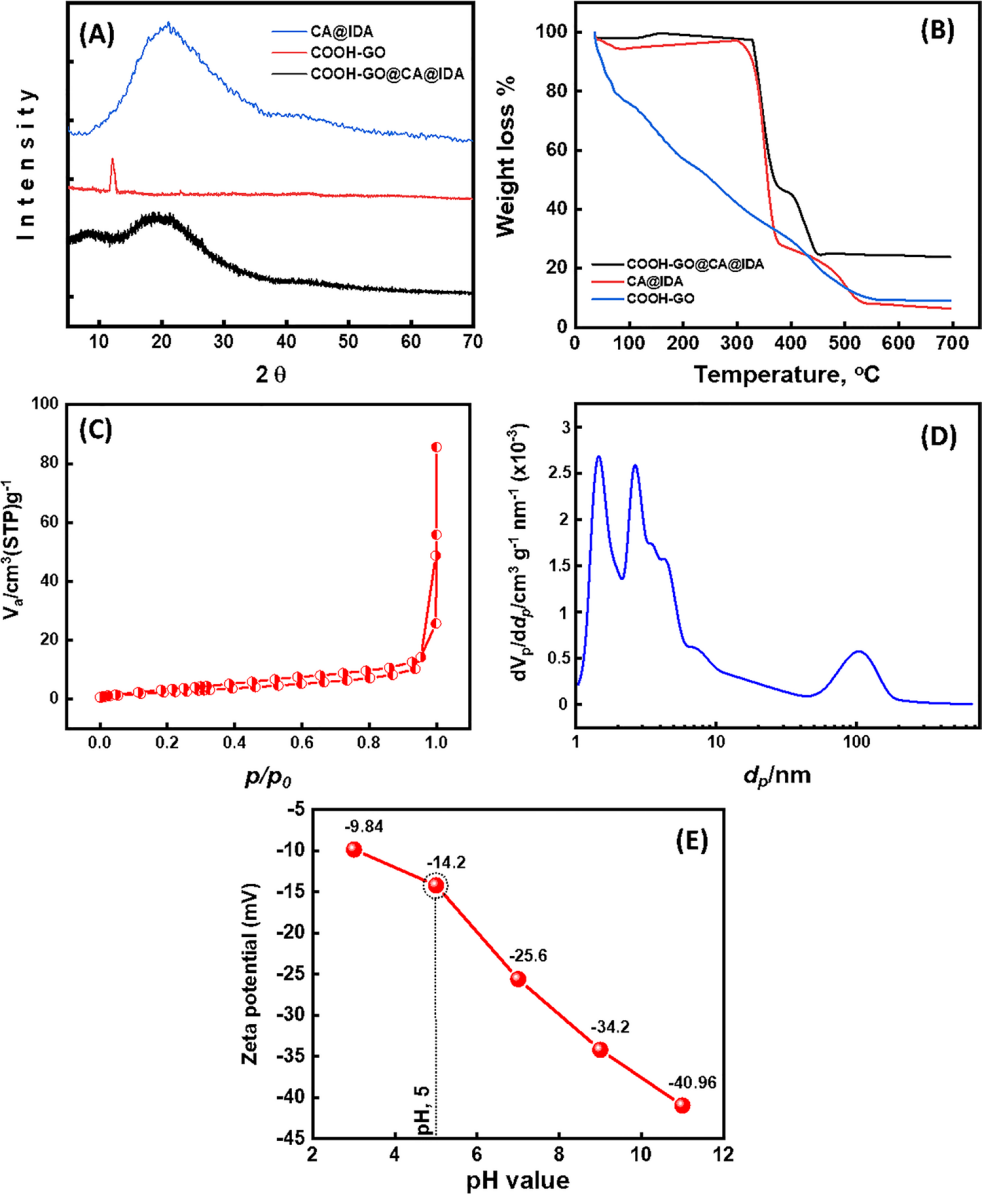
**A** XRD patterns. **B** TGA thermograms of CA@IDA, COOH-GO, and COOH-GO@CA@IDA spheres. **C** N_2_ adsorption–desorption isotherm. **D** BJH profile for pore size distribution of COOH-GO@CA@IDA sample. **E** Zeta potential of COOH-GO@CA@IDA

X-ray diffraction analysis was done to examine the crystallinity/amorphous nature and structural features of CA@IDA, COOH-GO, and COOH-GO@CA@IDA. The XRD patterns of three samples were investigated, as shown in Fig. 3A. The XRD spectrum of CA@IDA showed the diffraction peak at 2*θ* value of 21°, which confirms the presence of cellulose acetate matrix. CA@IDA is usually considered a semicrystalline polymer. The reason for the low crystallinity in CA@IDA is hydrogen bonding between -COOH and -OH groups. On the other hand, the XRD pattern of COOH-GO shows an intense and sharp peak centered at 12° (at reflection plane (001), with an interplanar distance of ~ 0.8 nm) which confirms the existence of oxygen functional groups which belong to GO matrix and -COOH groups. In the composite samples, the XRD pattern shows the major diffraction peak at 2*θ* = 8° corresponds to the carbon structure of COOH-GO. Additional diffraction peak around 2*θ* = 20° corresponds to CA@IDA. This observation indicates that the COOH-GO is well dispersed throughout the CA@IDA spheres matrix without forming aggregates. The obtained results indicate the successful formation of COOH-GO@CA@IDA spheres.

#### TGA

TGA was employed to investigate the thermal stability of COOH-GO@CA@IDA spheres, as shown in Fig. 3B. The thermograms indicate the weight loss of the COOH-GO, CA@IDA, and COOH-GO@CA@IDA adsorbent as a function of temperature. TGA curve showed that COOH-GO sample (blue line) degrade mainly in three steps, which show the high degree of oxidation and rich -COOH groups. The first step (36–105 °C) of ~ 25% mass loss belongs to the evaporation of adsorbed water which is located between the lamellar layers of COOH-GO. The major mass loss is between 110 and 330 °C by ~ 65%. In this range, decomposition occurs with the evolution of water molecules from hydroxyl groups, and carbon dioxide and carbon monoxide from carboxylic groups. In comparison with these results, CA@IDA, and COOH-GO@CA@IDA samples are stable at this stage, where about 3 and 2% were lost. Figure 3 also shows that about 72 wt% of CA@IDA were decomposed at around 377 °C. On the other hand, about 50 wt% of COOH-GO@CA@IDA was lost at around 377 °C. And third step starts after 377 °C and shows the carbonization of samples. After grafting of COOH-GO and CA@IDA to produce COOH-GO@CA@IDA, it is observed from the TGA curve that the thermal stability increases. The finding data indicated that COOH-GO@CA@IDA has more thermal stability than COOH-GO and CA@IDA (Abd El-Monaem et al. 2022b; Eltaweil et al. 2020a).

#### N_2_ adsorption–desorption isotherm

N_2_ adsorption–desorption isotherm was performed to assess the surface areas, pore size, and volume of the COOH-GO@CA@IDA sample based on Brunauer–Emmett–Teller (BET) theory. Figure 3C signifies that the isotherm models of COOH-GO@CA@IDA adsorbent followed a type-IV isotherm, where the adsorption/desorption curve was obtained at a P/Po of 0.3–0.98. The obtained curve indicated that COOH-GO@CA@IDA adsorbent was mesoporous and had specific surface areas of 9.6 m^2^/g and pore volumes of 0.036 cm^3^/g. The Barrett-Joyner-Halenda (BJH) diagram (Fig. 3D) indicated that the average pore diameter of the COOH-GO@CA@IDA sample was about 21.145 nm. The mesoporosity of the COOH-GO@CA@IDA adsorbent indicated (i) the presence of large numbers of surface active sites inside the interior pores; (ii) the facile diffusion of Pb(II) ions across the surface of applied adsorbent; and (iii) the enhanced trapping of Pb(II) ions inside pores, leading to high adsorption capacity (mg/g) (Gomaa et al. 2023c, d).

#### Zeta potential measurements

To determine the surface charge of COOH-GO@CA@IDA spheres, the zeta potential was measured. Figure 3E shows the zeta-potential analysis of the COOH-GO@CA@IDA spheres adsorbent in the range of pH 3–11. At pH 3–5, the exterior surface-active sites have a negative charge due to the plenty of -COOH groups on the surface, leading to enhance the trapping and adsorption of Pb(II) ions as positively charged species. With the pH further increasing, there were more negatively charged sites on the surface of COOH-GO@CA@IDA spheres because of the available hydroxyl ions.

### The influence of different adsorption factors

#### Synergistic effect between inner/outer modifications

**Fig. 4:**
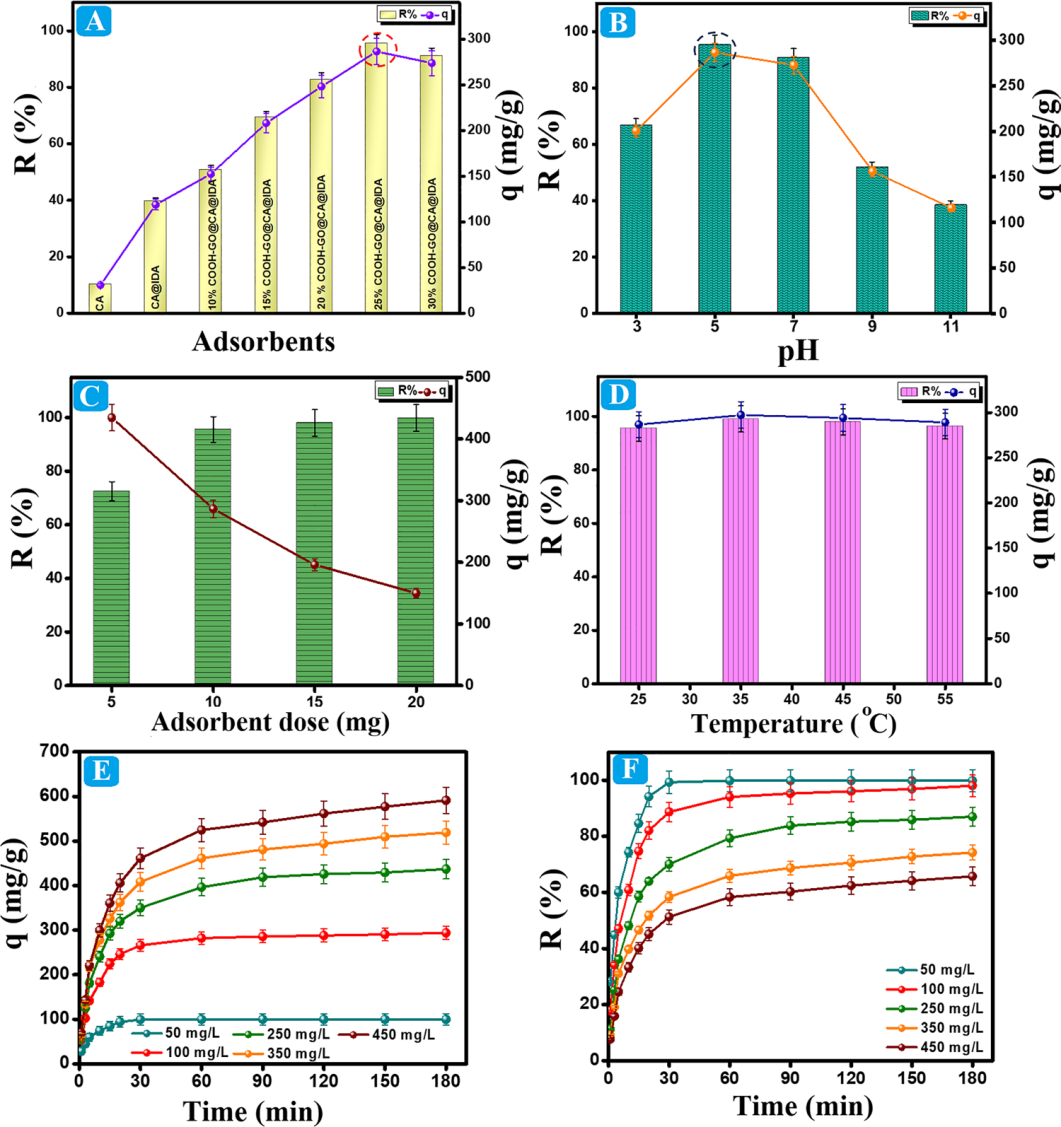
The influence of **A** COOH-GO proportions, **B** pH of the adsorption system, **C** COOH-GO@CA@IDA dose, **D** temperature of the adsorption system, and **E**, **F** the initial Pb(II) concentrations

Figure 4A clarifies the drastic improvement in the adsorption profile of CA spheres after the dual modifications. Firstly, the outer modification by attaching the carboxyl-containing IDA on the surface of the CA spheres enhanced the adsorption profile of CA spheres about fourfold where the R% of CA and CA@IDA toward Pb(II) were 10.24 and 39.62%, while *q* was 30.73 and 118.86 mg/g, respectively. Secondly, the inner modification by encapsulating COOH-GO onto CA@IDA furtherly incremented the R% and *q* of COOH-GO@CA@IDA sphere toward Pb(II) from 50.80% and 152.42 mg/g to 95.52% and 286.56 mg/g by raising the COOH-GO proportions from 10 to 25 wt%, subsequently. This observation may be attributed to increasing the carried negative charges inside and outside the spheres, which raises the tendency of the spheres to attract the Pb(II) species from the aqueous solution. The former findings implied controlling the electrostatic interactions on the COOH-GO@CA@IDA–Pb(II) system. Nevertheless, the over-incrementing in the COOH-GO amount to 30 wt% caused a pore-blocking and dwindled the R% and *q* of Pb(II) to 91.22% and 273.67 mg/g, respectively, agreeing with Eltaweil and his co-authors (Eltaweil et al. 2020a). Consequently, the experimental results denoted that 25 wt% was selected as the optimal encapsulation ratio of COOH-GO into CA@IDA. Furthermore, a synergistic effect was noticed between the inner and outer modification ways to enhance the adsorbability of CA spheres.

#### Influence of the pH solution

The pH solution of Pb(II) affects the adsorption performance in the COOH-GO@CA@IDA–Pb(II) system, as illustrated in Fig. 4B. The results show an enhancement in the adsorption aptitude of Pb(II) species by elevating the pH solution since the q was ameliorated from 200.27 to 286.56 mg/g and R% was enhanced from 66.85 to 95.52% by increasing the adjusted pH from 3 to 5. This observation has many reasons: (i) the increase in the amounts of the carried negative charges onto COOH-GO@CA@IDA as elucidated by the ZP measurements, and thus the capability of the spheres to grasp the Pb(II) species by the electrostatic interactions increased. (ii) At the strongly acidic medium (pH = 3), there are higher amounts of the protons that compete with Pb(II) for the negatively oxygenated adsorption sites of the spheres; therefore, the available binding sites to Pb(II) diminished. (iii) At pH 5, the distributed protons in the adsorption system declined, and the active oxygenated groups of the spheres comprising epoxy, carboxyl, and hydroxyl were ionized. Nonetheless, it was noticed a slight diminution in the adsorption% inside the COOH-GO@CA@IDA–Pb(II) system when raising pH to 7, followed by a dramatic decline over pH 9. This finding could be explained by the production of other Pb(II) species with lower positive charges, like Pb(OH)^+^ and Pb(OH)_2_, which weakened the electrostatic interaction between the spheres and Pb(II) species (Omer et al. 2023a). In addition, the participation of Pb(II) at high alkaline adsorption media. Thus, the optimal pH for the COOH-GO@CA@IDA–Pb(II) system was pH = 5.

#### Influence of the dose of COOH-GO@CA@IDA

Figure 4C shows the influence of the COOH-GO@CA@IDA dosage on the adsorption aptitude of Pb(II). The adsorption efficacy of Pb(II) diminished from 434.62 to 149.77 mg/g associated with elevating the weight of the used COOH-GO@CA@IDA from 5 to 20 mg at the same Pb(II) concentration. This observation may be explained by the increase in the unoccupied binding sites on the spheres’ surface. However, this increase in the spheres’ weight boosted the R% (72.43–99.84%) owing to the ample active functional groups that are available to adsorb Pb(II) (Abd El-Monaem et al. 2023; Abdelfatah et al. 2021; Liu et al. 2023).

#### Influence of the system temperature

The thermodynamic profile of the COOH-GO@CA@IDA–Pb(II) system was studied at a temperature range of 25–55 °C, as shown in Fig. 4D. An increase in the R% and q of Pb(II) onto COOH-GO@CA@IDA from 95.52% and 286.56 mg/g to 99.07% and 297.19 mg/g when increasing the temperature of the adsorption system from 25 to 35 °C owes to the increment in the Brownian motion of Pb(II) inside the bulk aqueous solution, which facilitates the Pb(II) adsorption. However, the increase in the system temperature over 35 °C resulted in a decline in the R% and *q* to 96.34% and 289.01 mg/g, subsequently. This observation is most probably due to the further increment in the kinetic energy of Pb(II) species that leads to the desorption of the ions out of the spheres (Swathanthra &Rao 2014).

#### Influence of the initial Pb(II) concentration

As elucidated in Fig. 4E, the *q* of Pb(II) fostered from 99.78 to 591.34 mg/g when the Pb(II) concentrations increased from 50 to 450 mg/L, which may be explained by the strengthening in the Pb(II) driving forces toward COOH-GO@CA@IDA, outdoing the resistance forces to the ions transfer. Conversely, this increment in the Pb(II) concentrations dwindled the R% from 99.78 to 65.71% (Fig. 4F) because of the inadequacy in the active binding sites for these high concentrations of Pb(II) (Abdelfatah et al. 2022). Notably, the adsorption process attained equilibrium within 20 min, and the removal% reached almost 100% after 30 min at the low Pb(II) concentration. While, at the high Pb(II) concentrations, the equilibrium time expanded to 90 min.

### ANOVA analysis

**Fig. 5:**
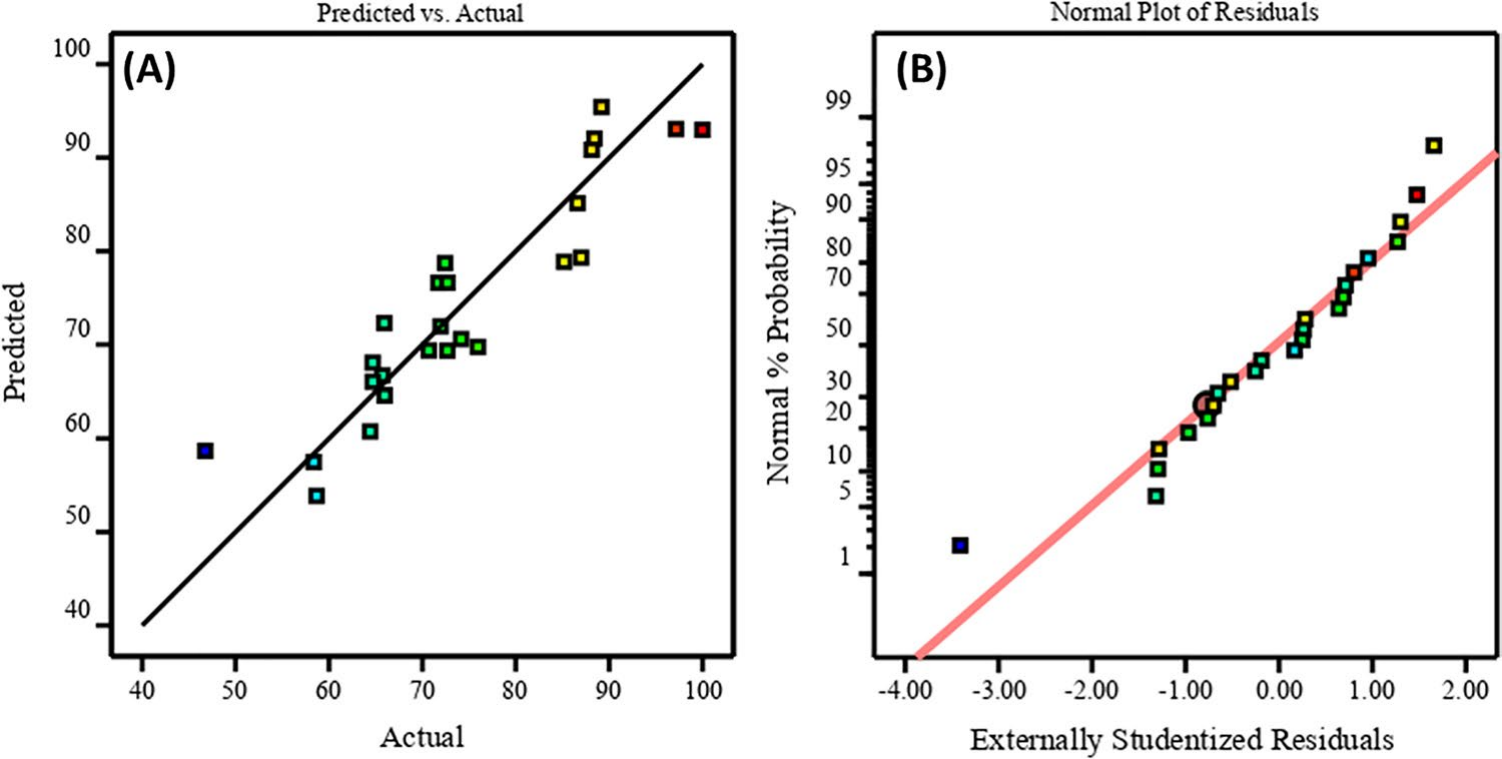
The plot of the relationship between predicted and experimental Pb(II)-removal % (**A**) and the normal probability plots of residuals obtained by ANOVA for Pb(II) removal using the COOH-GO@CA@IDA sample (**B**)

The essential role of the Design Expert software was evident in facilitating batch experiments focused on removing Pb(II) ions. These experiments employed response surface methodology within a 4-factors/3-level Box-Behnken design system, which generates designs with pertinent statistical features. The examined parameters in the batch system included pH (*A*), adsorbent dose (*B*), temperature (*C*), and initial Pb(II) concentration (*D*), as detailed in Table 1. The analysis of variance (ANOVA) statistics for Pb(II) removal using the COOH-GO@CA@IDA adsorbent is presented in Table 2. The *F*-values in Table 2 indicate that the models utilized for Pb(II) removal are adequate, with a significant *F*-value of 4.02 (*p*-value = 0.0163), indicating agreement between empirical and computed data. Parameters with *p*-values exceeding 0.05 are generally considered statistically insignificant in influencing Pb(II) removal performance. Therefore, parameters *A*, *B*, and *D* in the Pb(II) removal model were deemed statistically related. The final equation for the model using the COOH-GO@CA@IDA adsorbent, based on the quadratic model, is expressed as Pb(II) removal % = 74.36 + 5.36A + 6.01B – 11.78D. Figure 5A and B visually depict observed versus expected Pb(II)-removal values using principal components, demonstrating the model’s capability to replicate experimental outcomes.

**Table 1:** Coded symbols and ranges for batch mode parameters through BBD statistical experiment

**Variables design**	Factors	levels		
		** − 1**	**0**	** + 1**
***A***	pH	3	5	7
***B***	Adsorbent dose (mg)	5	12.5	20
***C***	Temperature (°C)	25	40	55
***D***	Initial Pb(II) concentration (mg/L)	50	200	350

**Table 2:** ANOVA results of the reduced quadratic model for Pb(II) removal using COOH-GO@CA@IDA sample

Source	Sum of squares	df	Mean square	*F*-value	*p*-value	
Model	3409.10	14	243.51	4.02	0.0163	Significant
*A*-pH	345.29	1	345.29	5.70	0.0382	
*B*-dose	433.44	1	433.44	7.15	0.0233	
*C*-temperature	13.31	1	13.31	0.2197	0.6494	
*D*-conc	1665.46	1	1665.46	27.48	0.0004	
AB	12.04	1	12.04	0.1986	0.6653	
AC	54.46	1	54.46	0.8985	0.3655	
AD	8.38	1	8.38	0.1383	0.7178	
BC	0.0000	1	0.0000	4.124E − 07	0.9995	
BD	95.94	1	95.94	1.58	0.2369	
CD	1.63	1	1.63	0.0268	0.8732	
*A*^2^	43.80	1	43.80	0.7227	0.4152	
*B*^2^	3.04	1	3.04	0.0501	0.8273	
*C*^2^	1.56	1	1.56	0.0257	0.8759	
*D*^2^	242.31	1	242.31	4.00	0.0735	
Residual	606.15	10	60.61			
Cor total	4015.24	24

### The supposed adsorption mechanism

#### Isotherm study

**Fig. 6:**
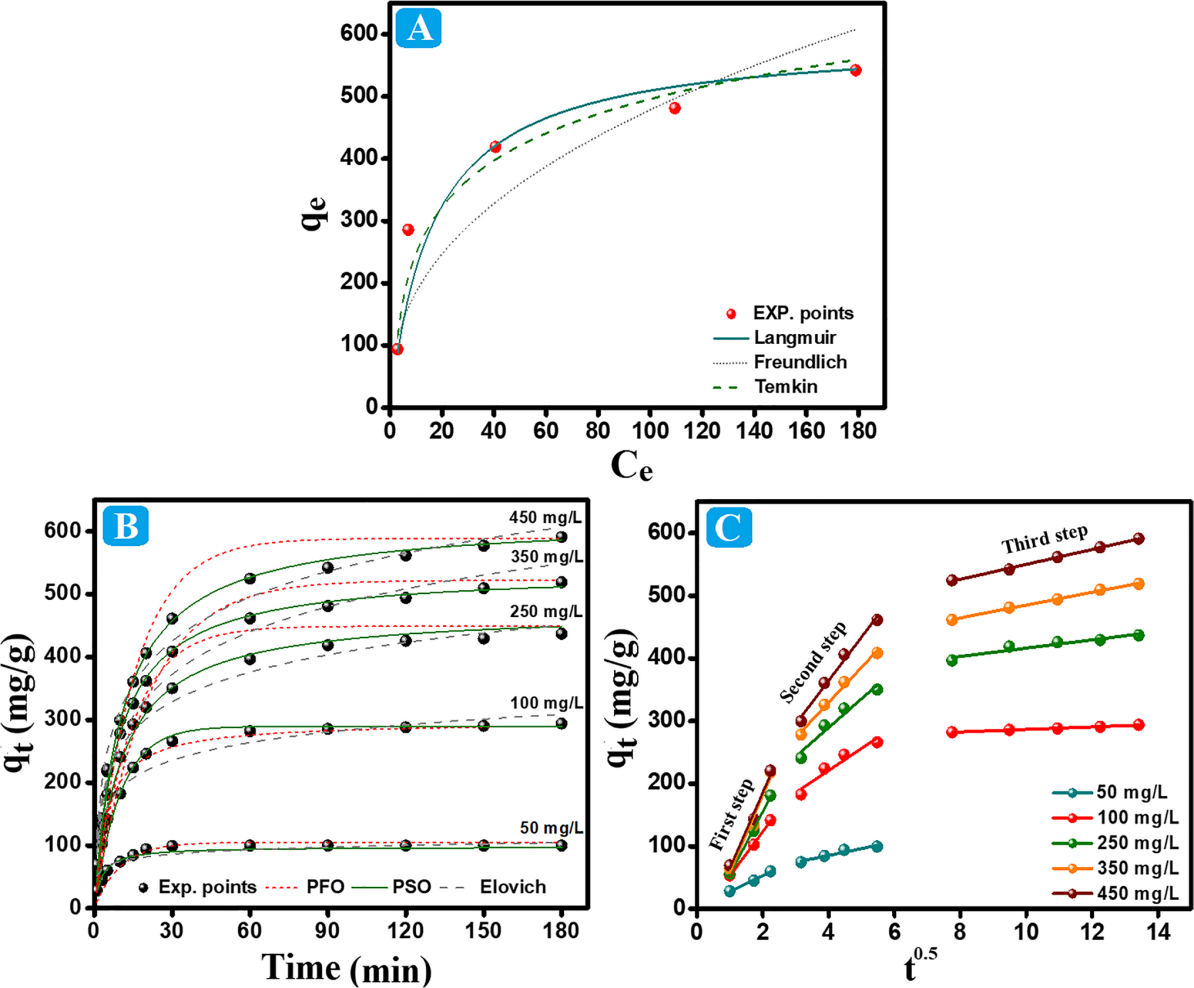
**A** Isotherms curves, **B** kinetics curves, and **C** intraparticle diffusion of the Pb(II) adsorption onto COOH-GO@CA@IDA spheres

To assess whether the adsorption process inside the COOH-GO@CA@IDA–Pb(II) system is dominated by physical or chemical interactions, Langmuir, Temkin, and Freundlich (Fig. 6A) modeled the obtained experimental data. Table S1 compiled the nonlinear isotherms expressions of these models.

Typically, Langmuir postulates the proceeding of the monolayer chemisorption mechanism inside adsorption systems (Eltaweil et al. 2023a). As shown in Table 3, the *R*^2^-Langmuir was the highest value (R2 = 0.804), indicated obeying the Pb(II) adsorption onto COOH-GO@CA@IDA to Langmuir model. The adsorption favorability could be confirmed by *R*_L_, where the adsorption is a linear process (*R*_L_ equals unity), irreversible (*R*_L_ equals zero), favorable (*R*_L_ is less than unity), and unfavorable (*R*_L_ is larger than unity) (Loganathan et al. 2022). The computed *R*_L_ values **(**Table S2**)** implied the Pb(II) adsorption favorability on the synthesized spheres since all the values are less unity (Dada et al. 2012). Notably, the maximal *q* was calculated from Langmuir to be 613.30 mg/g. Freundlich assumed the occurrence of adsorption processes via the multilayer physisorption pathways (Foroutan et al. 2021). The *R*^2^-Freundlich signified the inappropriateness of Freundlich to represent the Pb(II) adsorption onto COOH-GO@CA@IDA. The obtained n value from Freundlich was in the range of 1–10, implying the heterogeneity of the Pb(II) adsorption (Chakraborty et al. 2011).

**Table 3:** Parameters of Langmuir, Freundlich, Temkin, and D-R isotherms for the adsorption of Pb(II) ions onto COOH-GO@CA@IDA spheres

Isotherm model	Parameters	Value
Langmuir	*q*_max_ (mg/g) *K*_L_ (L/mg) *R*^2^	613.30 0.073 0.918
Freundlich	*n* *K*_f_ *R*^2^	2.43 71.95 0.822
Temkin	*A* (L/g) *b* (KJ/mol) *R*^2^	1.00 23.05 0.953

Temkin model could discriminate even the controlling interactions inside the COOH-GO@CA@IDA–Pb(II) system are physical or chemical based on *b* value (Wang et al. 2019). When b exceeds 80 kJ/mol, the Pb(II) adsorption process could proceed throughout the chemical interactions between Pb(II) species and the spheres, while the process could be physisorption if *b* is less than 80 kJ/mol (Erhayem et al. 2015; Gomaa et al. 2023a). The derived b value was 23.05 kJ/mol, evinced the occurrence of the adsorption process by physical interactions between the active binding groups of COOH-GO@CA@IDA spheres and Pb(II) ions.

#### Kinetic study

Kinetics study was performed by analyzing the experimental results using Pseudo-1st order (PFO), Pseudo-2nd order (PSO), intraparticle diffusion, and Elovich models (Fig. 6B, C). The equations of nonlinear models were summarized in Table S3.

The PFO assumes that physical interactions are responsible for occurring the adsorption process (Chakraborty et al. 2011). By modeling the experimental results in the PFO, it was found that the R^2^ values were lower than in the PSO. Additionally, the calculated equilibrium adsorption capacities at the varied Pb(II) concentrations were not closer to the experimental values. Hence, the PFO model is inappropriate to represent the adsorption process inside the COOH-GO@CA@IDA–Pb(II) system. Contrariwise, the PSO proposes the chemical interactions as the possible pathways for proceeding with the adsorption processes (Gomaa et al. 2023b; Wang et al. 2019). Notably, the larger *R*^2^ of PSO and the closer computed adsorption capacities of Pb(II) at the different concentrations to the experimental data indicated the fitness of PSO to model the Pb(II) adsorption onto COOH-GO@CA@IDA (Table 4).

**Table 4:** PFO, PSO, and Elovich models parameters for adsorption of Pb(II) ion onto COOH-GO@CA@IDA spheres

Kinetic models and parameters	Concentration (mg/L)				
	**50**	**100**	**250**	**350**	**450**
*q*_e,exp._ (mg/g)	94.15	285.81	418.86	481.09	542.1
PFO					
*q*_e,cal._ (mg/g)	104.69	299.68	479.07	522.32	588.97
*k*_1_ (min^−1^)	0.100	0.087	0.072	0.050	0.065
*R*^2^	0.823	0.951	0.945	0.901	0.962
PSO					
*q*_e,cal._ (mg/g)	98.01	289.72	449.95	538.28	621.34
*k*_2_ (g. mg^−1^. min^−1^)	0.0037	0.001	0.00026	0.0002	0.0001
*R*^2^	0.954	0.979	0.988	0.995	0.996
Elovich					
*α* (mg/g min)	1577.58	436.26	279.75	137.91	218.36
*β* (g/mg)	0.0982	0.0245	0.0147	0.0100	0.0098
*R*^2^	0.803	0.907	0.954	0.976	0.971

Similar to the PSO, Elovich presumes controlling the chemisorption mechanism on adsorption systems, postulating the heterogeneous surface, and raising the activation energy with the contact time (Eltaweil et al. 2022). Noteworthy, the high values of *R*^2^ denoted the role of the chemical interactions in adsorbing Pb(II) onto COOH-GO@CA@IDA. It is essential to ensure the irreversibility of the adsorption process since the rate of pollutants adsorption should be higher than their desorption rate. The derived *α* and *β* values assured the higher Pb(II) adsorption rate onto the spheres than the desorption rate, inferring the irreversibility and favorability of the Pb(II) adsorption process (Basha et al. 2022).

Intraparticle diffusion estimates the migration steps of the Pb(II) species from their solution to the COOH-GO@CA@IDA spheres. The intraparticle diffusion plot depicts the Pb(II) diffusion pathway that occurred throughout two steps at the low concentration and three steps at the high concentrations of Pb(II). (i) The Pb(II) species gradually emigrated from the bulk solution to adsorb onto the surface of COOH-GO@CA@IDA in the first step. (ii) In the second step, the Pb(II) species diffused through the COOH-GO@CA@IDA pores and reached equilibrium in the low concentration. (iii) For the high Pb(II) concentration, the ions penetrated through the interior pores of COOH-GO@CA@IDA spheres at the third step until attained equilibrium.

As elucidated in Table 5, *K*_p_ of the first step > second > third owes to changing the Pb(II) diffusion rate throughout the adsorption steps. In addition, *K*_p_ increased by elevating the Pb(II) concentrations, which is most probably due to strengthening the driving force by raising the Pb(II) concentrations. Furthermore, the intraparticle diffusion curves did not pass through the origin, indicating that the intra-particle diffusion is not only the rate-controlling step.

**Table 5:** The parameters of the intraparticle diffusion kinetic model

Co	First step			Second step			Third step		
	*K*_p,1_	*C*_1_	*R*^2^	*K*_p,2_	*C*_2_	*R*^2^	*K*_p,3_	*C*_3_	*R*^2^
50	25.49	2.13	0.988	10.89	41.79	0.908	-	-	-
100	70.97	18.52	0.998	35.26	80.01	0.899	2.03	266.23	0.988
250	100.58	45.91	0.996	46.21	104.72	0.927	6.61	350.33	0.893
350	122.82	64.93	0.949	56.12	105.44	0.987	10.20	383.51	0.996
450	126.98	75.92	0.972	69.65	108.53	0.981	11.93	430.93	0.997

The intraparticle diffusion findings clarified that the adsorption of low concentration of Pb(II) occurred on the outer surface of the spheres and their pores without entering the interior pores. This result explained reaching the adsorption process of low Pb(II) concentration to equilibrium during 20 min. On the contrary, in the higher concentrations of Pb(II), the adsorbed Pb(II) species reached the interior pores of the spheres, so the equilibrium time expanded to 90 min.

### XPS of COOH-GO@CA@IDA spheres

The isotherms study denoted controlling the physical interactions on the COOH-GO@CA@IDA–Pb(II) adsorption system, while the kinetic study implied the occurrence of the chemical interactions between Pb(II) and the spheres. For identifying how the chemical and physical interactions attack Pb(II) species to the active adsorption sites of COOH-GO@CA@IDA spheres, XPS of the authentic and used spheres were studied. Table S4 summarizes the compositional percentages of COOH-GO@CA@IDA spheres before and after the Pb(II) adsorption.

**Fig. 7:**
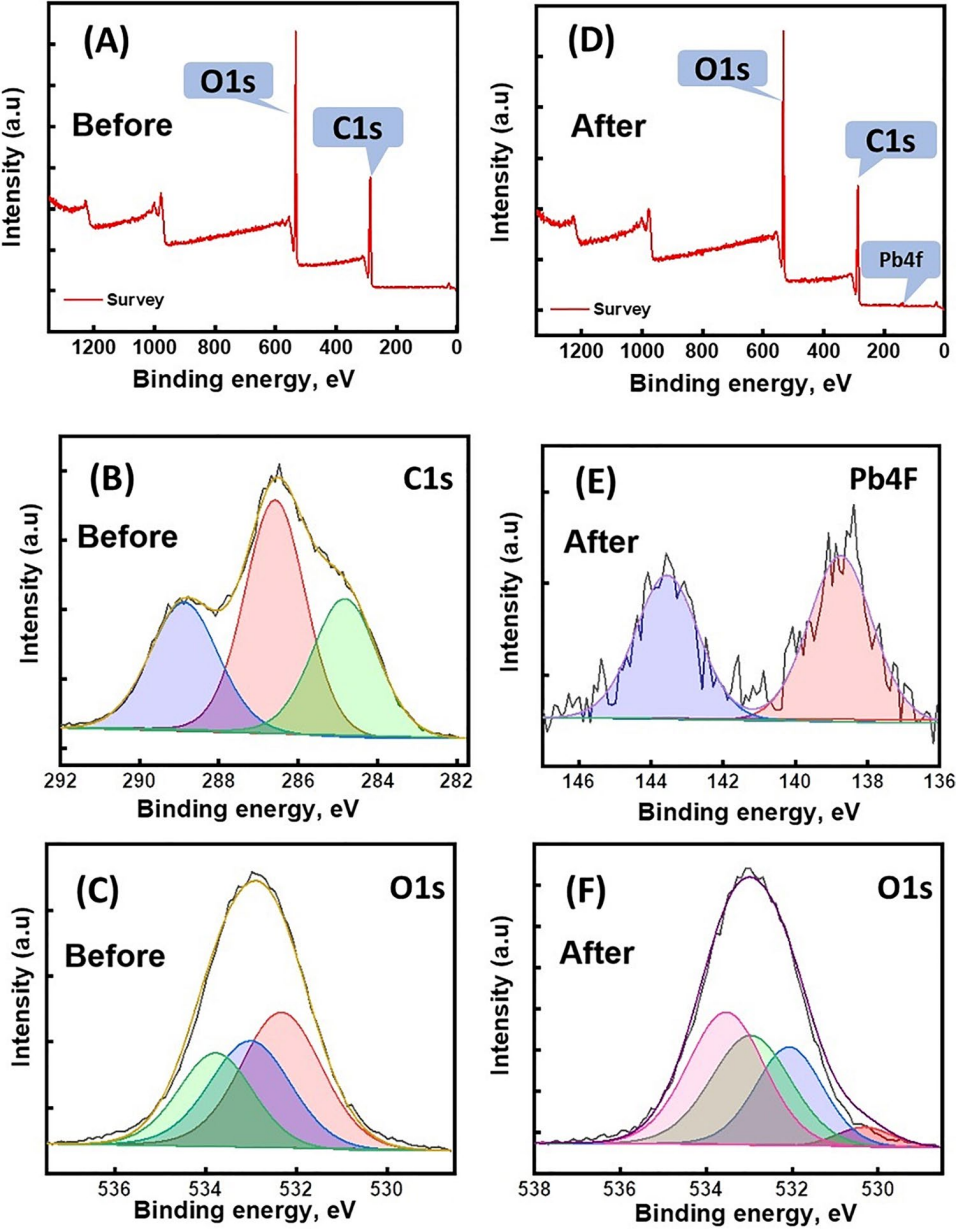
The XPS spectra of COOH-GO@CA@IDA spheres: **A**–**C** before and **D**–**F** after the Pb(II) adsorption

The wide scan of the authentic COOH-GO@CA@IDA depicts the composing of the spheres from C1s and O1s with atomic percentages of 62.43 and 37.57%, respectively (Fig. 7A). The C1s-high resolution (Fig. 7B) deconvoluted into three subpeaks at 284.8, 286.5, and 288.8 eV which are assigned to C–C, C–O–C, and O = C–O, subsequently. Additionally, the O1s-high resolution built from three subpeaks at 532.3, 533.0, and 533.8 eV which are ascribed to C–O, C–OH, and O = C–O, as shown in Fig. 7C.

The wide scan of the used COOH-GO@CA@IDA spheres illustrated a new XPS peak at 139.8 eV which corresponded to Pb4f, indicating the successful trapping of Pb(II) ions by COOH-GO@CA@IDA (Fig. 7D). The belonging spectrum of Pb4f elucidated two subpeaks of (Pb–O)4f7/2 at 138.75 eV and (Pb–O)4f5/2 at 143.56 eV (Fig. 7E). The O1s-high resolution of Pb(II)-adsorbed COOH-GO@CA@IDA revealed the related peak to P-O at 530.3 eV with a redshift, reflecting the bond formation between Pb(II) species and the oxygenated binding sites of the spheres (Fig. 7F). The OH and COOH groups of COOH-GO@CA@IDA could grasp the Pb(II) species by forming Lewis acid–base interactions. In addition, these active oxygenated groups could adsorb Pb(II) by a complexation mechanism (Omer et al. 2023a). Noteworthy, the precipitation mechanism can participate in removing the Pb(II) species by forming lead hydroxide. Hence, we could deduce that the complexation, Lewis acid–base, and precipitation are the chemical pathways to adsorb Pb(II) onto COOH-GO@CA@IDA spheres.

**Fig. 8:**
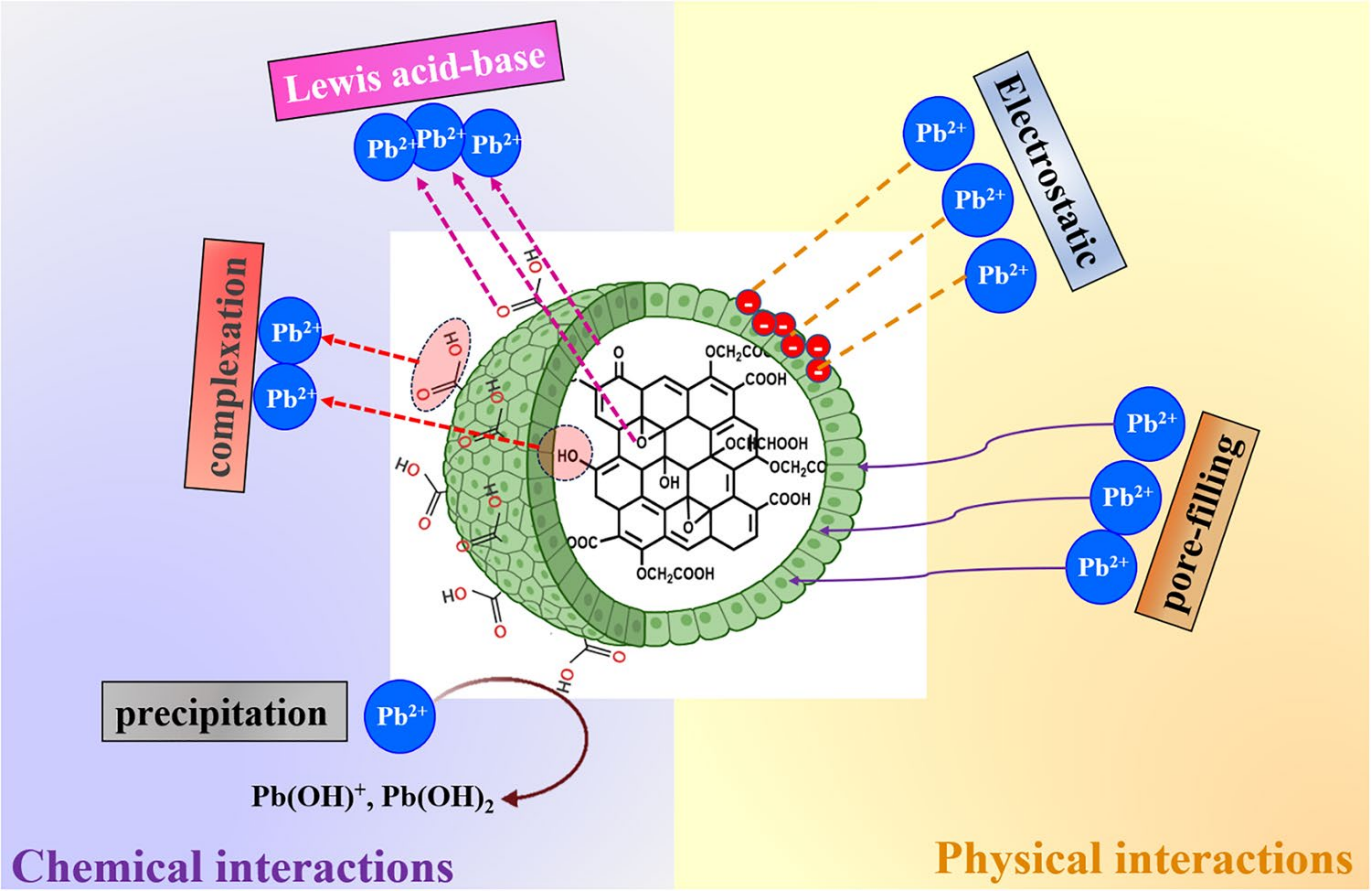
Schematic representation to the interactions inside the COOH-GO@CA@IDA–Pb(II) adsorption system

The experimental results and ZP measurements (zeta potential =  − 14.2 mV at pH = 5) asserted that the electrostatic interaction between the cationic Pb(II) species and the anionic binding groups of COOH-GO@CA@IDA spheres is the controlling physical pathway in this adsorption system. Additionally, the unique channel-like pores of the COOH-GO@CA@IDA spheres that was obviously appeared in SEM images rendered the pore-filling mechanism of Pb(II) possible. Figure 8 elucidates the interactions inside the COOH-GO@CA@IDA–Pb(II) adsorption system.

In one word, the adsorption of Pb(II) onto COOH-GO@CA@IDA spheres occurred via a chemical pathway involving complexation, Lewis acid–base, and precipitation. In addition, the physical pathway comprised the electrostatic interactions between Pb(II) and the spheres and pore filling mechanism.

### Recyclability study

**Fig. 9:**
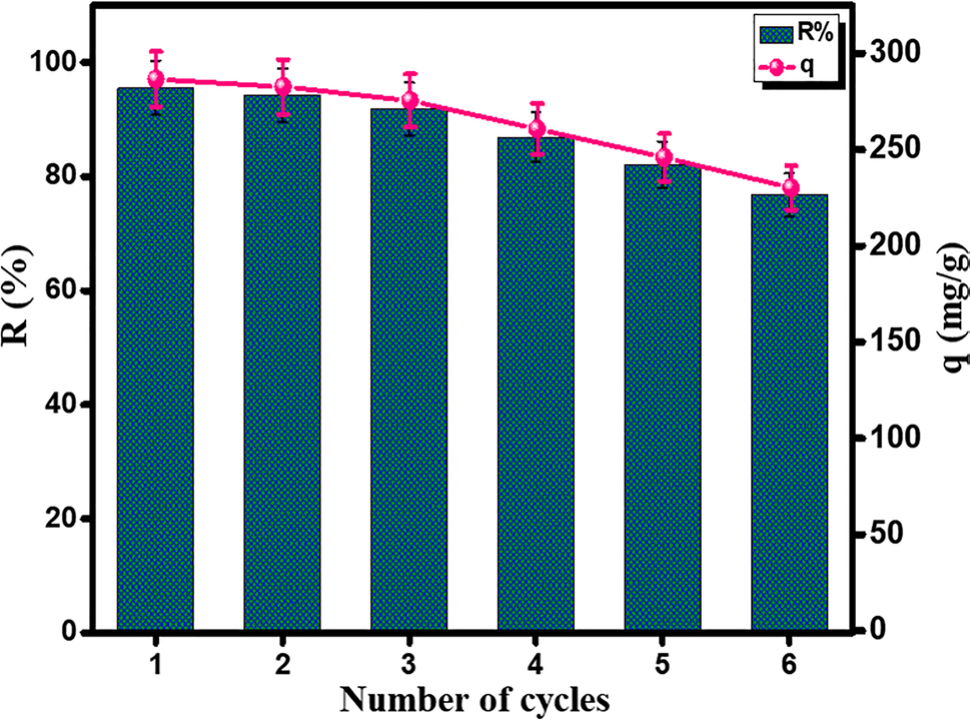
Recyclability study of COOH-GO@CA@IDA spheres in adsorbing Pb(II) for six cycles

The recyclability of COOH-GO@CA@IDA spheres was investigated by performing the cycling test for six runs (Fig. 9). The R% and q of Pb(II) declined from 95.52% and 286.56 mg/g to 76.74% and 230.21 mg/g during six adsorption runs. It was observed a slight diminution in the R% and q of Pb(II) through the first four cycles by 8.58% and 25.74 mg/g, respectively, denoting the excellent recyclability of COOH-GO@CA@IDA spheres. Nonetheless, the R% and q declined by 10.19% and 30.62 mg/g during the 5th and 6th cycles since the amounts of the encapsulated COOH-GO began to leach from the pores of the spheres. Hence, COOH-GO@CA@IDA spheres need improvements to be reusable for more than four cycles.

## Conclusion

An eminent adsorbent-based cellulose acetate was fabricated via dual inner and outer modifications for efficacious removal of Pb(II) species. The ZP measurements showed the abundance of negative charges onto the COOH-GO@CA@IDA spheres, reflecting their appropriateness to adsorb bountiful cationic contaminants. The SEM images revealed the wide channel-like pores of COOH-GO@CA@IDA, which is an individual character of the CA-based adsorbents that boosts their adsorption properties via the pore-filling mechanism. Interestingly, the adsorption process attained equilibrium within 20 min, and the removal% reached almost 100% after 30 min at the low Pb(II) concentration. Moreover, at the high Pb(II) concentrations, the equilibrium time expanded to 90 min with a maximal adsorption capacity of about 613.30 mg/g.

Despite, the remarkable adsorbability of COOH-GO@CA@IDA spheres, the equilibrium time at high concentrations is a bit long. Consequently, COOH-GO@CA@IDA spheres need investigations to decrease the equilibrium time, which may occur by encapsulating substances with fast adsorption merits, such as metal–organic frameworks and layered double hydroxide. These incorporated substances could also increase the negative charges on the surface of the spheres. Furthermore, COOH-GO@CA@IDA spheres exhibited outstanding recyclability during the first four cycles of the Pb(II) adsorption/desorption, followed by a bit drop in their reusability during the subsequent two cycles. Thus, we could recommend boosting the recyclability by coating the spheres with a polymeric material, avoiding the leaching of the encapsulated substances.

### Supplementary Information

Below is the link to the electronic supplementary material.

Supplementary file1 (DOCX 216 KB)

## Data Availability

The data sets used and analyzed during the current study are available from the corresponding author on reasonable request.
