# Supplementary material for: Sequestration of Pb(II) using channel-like porous spheres of carboxylated graphene oxide-incorporated cellulose acetate@iminodiacetic acid: optimization and mechanism study
Source: Environ Sci Pollut Res Int. 2024 Apr 25;31(22):32664–79. doi: 10.1007/s11356-024-33185-1 (PMC11133213; doi:10.1007/s11356-024-33185-1)
Supplement: Supplementary file 1 — Supplementary file1 (DOCX 216 KB) [file 11356_2024_33185_MOESM1_ESM.docx]

**Sequestration of Pb(II) using channel-like porous spheres of carboxylated graphene oxide-incorporated cellulose acetate@**i**minodiacetic acid: optimization and mechanism study**

**Eman M. Abd El-Monaem^1^, Hassanien Gomaa^2^, Ahmed M. Omer^3,^*, Gehan M. El-Subruiti^1^,** **Abdelazeem S. Eltaweil^1^**

^1^Chemistry Department, Faculty of Science, Alexandria University, Alexandria, Egypt.

^2^Department of Chemistry, Faculty of Science, Al-Azhar University, Assiut 71524, Egypt.

^3^Polymer Materials Research Department, Advanced Technology and New Materials Research Institute (ATNMRI), City of Scientific Research and Technological Applications (SRTA-City), P. O. Box: 21934, New Borg El-Arab City, Alexandria, Egypt.

***Corresponding authors: Ahmed M. Omer (ahmedomer_81@yahoo.com)**

**Text S1**

Cellulose acetate (Mwt = 30,000 g/mol, 39.8 wt% acetyl) and graphite powder (<20 µm, purity 99.99%) was provided from Alfa-Aesar Co. (UK). Lead nitrate (Pb(NO₃)₂), sulfuric acid (assay 95-98%), and potassium permanganate (assay >99%) were brought from Aladdin Industrial Corporation (China). Hydrochloric acid (assay 37%), iminodiacetic acid, and hydrogen peroxide (assay 30%) were purchased from Loba Chemie (India).

**Text S2**

The fabricated samples were characterized by fourier transforms infrared spectroscopy (FTIR- Bruker vector-22), thermogravimetric analyzer (TGA- LINSEIS L40/2052), scanning electron microscope (SEM- Hitachi S4800), X-ray photoelectron spectroscopy (XPS- Thermo-Fisher Sci.), X-ray diffraction (XRD- VG ESCALAB 210), and Zeta potential (ZP- Malvern).


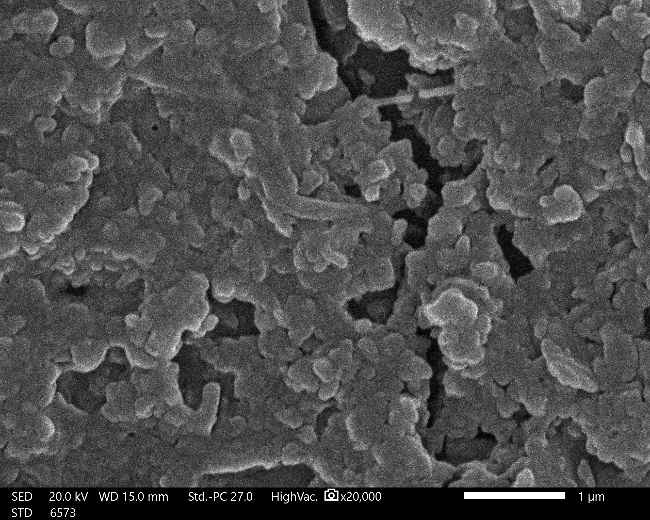


**Figure S1.** SEM image of COOH-GO sample.

**Table S1.** Non-linear equations of the applied adsorption isotherm models

| **Model** | **Equation** |
| --- | --- |
| **Langmuir** | $q_{e}=\frac{q_{max}K_{L}C_{e}}{1+ K_{L} C_{e}}$ (3) |
| **Freundlich** | $q_{e}=k_{F}C_{e}^{1/n}$ (4) |
| **Temkin** | $q_{e}=\frac{R T}{b_{T}}\ln k_{T}C_{e}$ (5) |

Where, q_e_ and C_e_ are the adsorption capacity and the concentration of the un-adsorbed Pb(II) at equilibrium, respectively. q_max_ and k_L_ are the monolayer adsorption capacity and Langmuir constant, respectively. n and k_F_ are Freundlich constants. k_T_ is the equilibrium binding constant and b is Temkin constant related to heat of adsorption.

**Table S2.** The calculated R_L_ values at varied concentrations of Pb(II).

| **C_o_ (mg/L)** | **R_L_** |
| --- | --- |
| 50 | 1.98x10^-4^ |
| 100 | 9.9x10^-5^ |
| 250 | 3.96x10^-5^ |
| 350 | 2.83x10^-5^ |

**Table S3.** Equations of the applied adsorption kinetic models

| **Kinetic Model** | **Equation** |
| --- | --- |
| **Pseudo 1^st^ order** | $q_{t}=q_{e}\left( 1-e^{-k_{1}t} \right) (7)$ |
| **Pseudo 2^nd^ order** | $q_{t}=\frac{tk_{2}q_{e}^{2}}{1+t k_{2}q_{e}} (8)$ |
| **Elovich model** | $q_{t}= \frac{1}{\beta}ln(\alpha\beta t+1)$ (9) |
| **Intraparticle diffusion** | $q_{t}=K_{p}t^{0.5}+C$ (10) |

Where, q_t_ and q_e_ are amounts of Pb(II) uptakes at time t and equilibrium, respectively. k_1_ and k_2_ are the rate constants of Pseudo-1^st^ order and Pseudo-2^nd^ order, respectively. Furthermore, α and β are Elovich coefficients that represent the initial adsorption rate and the desorption coefficient, respectively, also related to the extent of surface coverage and activation energy for chemisorption. K_p_ is the intraparticle diffusion constant and C provides an idea about the thickness of the boundary layer.

**Table S4.** XPS data of COOH-GO@CA@IDA spheres (before and after Pb^2+^-adsorption).

|  | C 1s | | | O 1s | Pb 4f |  |
| --- | --- | --- | --- | --- | --- | --- |
| Before Pb^2+^-adsorption | Survey data | | 62.43 C (%)  287.5 eV | 37.57 O (%)  533.7 eV | 0 Pb (%)  ---- |  |
|  | Peak positions | 288.8 eV  286.5 eV  284.8 eV | | 533.8 eV  533 eV  532.3 eV | ---- |  |
| After Pb^2+^-adsorption | Survey data | | 62.12 C (%)  287.4 eV | 37.72 O (%)  533.9 eV | 0.16 Pb (%)  139.8 eV |  |
|  | Peak positions | 288.8 eV  286.5 eV  284.8 eV | | 533.5 eV  532.9 eV  532 eV  530.3 eV | 138.75 eV  143.56 eV | |
